# Synthesis of Cu Nanoparticles Incorporated Mesoporous C/SiO_2_ for Efficient Tetracycline Degradation

**DOI:** 10.3390/nano13172478

**Published:** 2023-09-02

**Authors:** Ning Wang, Yuanyuan Zhao, Xuelian Wu, Dapeng Li, Ruguang Ma, Zhigang Chen, Zhengying Wu

**Affiliations:** 1 Jiangsu Key Laboratory for Environment Functional Materials, School of Materials Science and Engineering, Suzhou University of Science and Technology, Suzhou 215009, China; 2 School of Chemistry and Life Science, Suzhou University of Science and Technology, Suzhou 215009, China; 3 Jiangsu Collaborative Innovation Center of Technology and Material for Water Treatment, Suzhou University of Science and Technology, Suzhou 215009, China

**Keywords:** Cu nanoparticles, mesoporous silica, Fenton-like catalyst, tetracycline

## Abstract

In this study, a Cu NPs-incorporated carbon-containing mesoporous SiO_2_ (Cu/C-SiO_2_) was successfully synthesized through a grinding-assisted self-infiltration method followed by an in situ reduction process. The obtained Cu/C-SiO_2_ was then employed as a Fenton-like catalyst to remove tetracycline (TC) from aqueous solutions. TEM, EDS, XRD, N_2_ adsorption–desorption, FTIR, and XPS methods were used to characterize the crystal structure, morphology, porosity, chemical composition, and surface chemical properties of the catalyst. The effects of initial TC concentration, catalyst dosage, H_2_O_2_ dosage, solution pH, HA addition, and water media on the TC degradation over Cu/C-SiO_2_ were investigated. Scavenging and electrochemical experiments were then carried out to analyze the TC degradation mechanism. The results show that the Cu/C-SiO_2_ can remove 99.9% of the concentrated TC solution (*C*_0_ = 500 mg·L^−1^), and it can be used in a wide pH range (*R.E.* = 94–99%, pH = 3.0–11.0). Moreover, hydroxyl radicals (•OH) were detected to be the dominant reactive species in this catalytic system. This study provides a simple and promising method for the synthesis of heteroatom-containing mesoporous catalysts for the decomposition of antibiotics in wastewater.

## 1. Introduction

Antibiotics have been widely used in medical, animal husbandry, aquaculture industries, and so on. However, it has been revealed that only a small portion of antibiotics used in humans and animals are metabolized or absorbed in the body, and approximately 60% of consumed human and veterinary antibiotics are ultimately released as the parent compounds into the environment [1]. On the other hand, a large number of antibiotics have been discharged into the environment by medical institutions, pharmaceutical companies, aquaculture farms, and so on, which also causes serious pollution to the environment. These residual antibiotics in the environment were circulated and finally accumulated in water and soil, posing a threat to ecological balance and human health [2]. Therefore, it is necessary to find an effective and environmentally friendly way to deal with antibiotic pollutants.

Various techniques including adsorption [3], biodegradation [4], photocatalysis [5], electrochemical oxidation [6], and advanced oxidation processes (AOPs) [7] have been applied to remove antibiotics from wastewater. Among them, the AOPs based on free radicals are extremely popular due to their high activity and strong oxidation ability. The free radicals (e.g., hydroxyl radical •OH, superoxide radicals •O_2_^−^, or sulfate radical SO_4_^•−^) can efficiently degrade antibiotics in aqueous solutions [8]. Fenton reaction is a traditional AOPs technique that has attracted great and continuous interest for its low cost, high efficiency, and mild conditions [9]. However, traditional Fenton technology based on Fe(II) ions has limitations, such as being conducted under low acidic conditions, accumulating Fe-containing sludge, etc. [10,11]. To overcome these shortcomings, Fenton-like reactions which apply heterogeneous catalysts as alternatives to replace Fe(II) ions have been developed. 

In heterogeneous Fenton-like reactions, transition metals (Fe, Co, Cu, Mn, etc.) and their oxides, are considered the most effective catalysts and have been extensively studied [12,13,14,15]. Among them, Cu-based heterogeneous catalysts, which have similar redox properties to iron but a broader pH range than the Fe-based redox system, showed great potential in the degradation of antibiotics in aqueous solutions [14,15,16]. Moreover, the Cu-containing catalysts were also proved to have good adsorption capabilities, which could facilitate the in situ catalytic degradation of the pre-adsorbed pollutants [16]. However, the catalytic activity of pristine Cu or copper oxide is limited due to their less-exposed catalytic sites. Consequently, Cu species are normally dispersed or incorporated onto supports with larger surface areas and better chemical stabilities [14,15,16]. 

Mesoporous SiO_2_ that has high stability, large surface area, uniform pore size, and ordered mesoporous channels can be served as good catalyst support [17,18]. Many efforts have already been devoted to introducing active sites into the mesoporous SiO_2_ and applying them as Fenton-like catalysts. For example, a multi-valent Co species doped mesoporous SiO_2_ was prepared to eliminate refractory organic pollutants, showing activity and stability in a very wide pH range [19]. In our previous studies, Fe_2_O_3_ and Co_3_O_4_ nanoparticles were incorporated into mesoporous SiO_2_ and showed superior heterogeneous Fenton-like catalytic activity for the removal of organic pollutants from aqueous solutions [20,21]. Moreover, Cu-modified mesoporous materials including Cu/TUD-1 [22], Cu-MSMs [23], Au@Cu_2_O [24], TiO_2_-Au@Cu_7_S_4_ [25], and Cu@C/SiO_2_ NFMs [14] were also explored as Fenton-like catalysts to degrade various water pollutants. It is confirmed that loading Cu onto mesoporous SiO_2_ materials could dramatically improve their catalytic performance. Nevertheless, the correlations among the structure of Cu-loaded catalysts, the size and chemical state of Cu species, and its catalytic activity were still less-discovered up to now. 

In the present study, Cu nanoparticles (NPs) incorporated carbon-containing mesoporous SiO_2_ (Cu/C-SiO_2_) were feasibly synthesized using the template-containing mesoporous SiO_2_ and Cu(CH_3_COO)_2_·H_2_O as the precursors via a grinding-assisted self-infiltration approach. The Cu NPs were uniformly dispersed on the matrix of mesoporous SiO_2_ and stabilized by the in situ generated C in the mesopores. The finally obtained Cu/C-SiO_2_ was then used as a Fenton-like catalyst and performed excellent catalytic activity to degrade TC from aqueous solutions. Additionally, the effects of the initial concentration of TC, the dosage of catalyst and H_2_O_2_, the solution pH, the addition of humic acid (HA), and different water on the degradation efficiency towards TC were thoroughly investigated. Furthermore, the possible catalytic mechanism was carefully studied through radical trapping experiments. This study has provided a flexible way of fabricating nano-metal-modified mesoporous materials for the efficient degradation of antibiotics from wastewater.

## 2. Materials and Methods

### 2.1. Chemicals and Reagents

Tetraethyl orthosilicate (TEOS, Mw = 208.33), aluminum chloride hexahydrate (AlCl_3_·6H_2_O, Mw = 241.43), copper acetate monohydrate (Cu(CH_3_COO)_2_·H_2_O, Mw = 199.65), hydrochloride (HCl, Mw = 36.46), and sodium hydroxide (NaOH, Mw = 40.00) were supplied by Sinopharm Chemical Reagent (Shanghai) Co., Ltd. of China. Pluronic P123 (EO_20_PO_70_EO_20_, Mw = 5800) was purchased from Sigma-Aldrich. Tetracycline hydrochloride (C_22_H_24_N_2_O_8_·HCl, TC, Mw = 480.90), and humic acid (HA) were purchased from Aladdin reagent (Shanghai) Co., Ltd. of China. All chemicals and reagents are of analytical grade and were used without further purification.

### 2.2. Synthesis of the Mesoporous Cu-C/SiO_2_

The template-containing mesoporous SiO_2_ was synthesized by a hydrothermal method [26]. Typically, 2.0 g of P123 and 4.82 g of AlCl_3_·6H_2_O were dissolved in 75 mL of deionized water, and the clear solution was subsequently heated to 35 °C. Then, 4.16 g of TEOS was added to the solution, which was continuously stirred at 35 °C for 24 h. After that, the mixture was further hydrothermally treated in an autoclave at 100 °C for another 24 h. Finally, the white solid product was filtered, washed, and dried. The P123 template in the sample was measured to be 31 wt% by a TG-DSC analysis (Figure S1).

The Cu NPs incorporated carbon-containing mesoporous SiO_2_ were prepared by a simple grinding-assisted self-infiltration method followed by an in situ carbonization reduction process. Typically, 0.6087 g of template-containing mesoporous SiO_2_ (7 mmol) and 0.2795 g Cu(CH_3_COO)_2_·H_2_O (1.4 mmol) were ground to obtain a homogeneous powder mixture, which was labeled Cu/C-SiO_2_-AS. Then, the mixture was treated at 550 °C for 2 h under N_2_ atmosphere, and the finally obtained material was named Cu/C-SiO_2_.

### 2.3. Characterizations

Characterizations of the materials are elucidated in Supporting Information, Text S1.

### 2.4. Catalytic Experiments

Catalytic experiments were carried out in a 250 mL glass conical beaker containing 150 mg of catalyst and 150 mL of TC solution (500 mg L^−1^) at room temperature (20 ± 2 °C). After the adsorption for 2 h, 15 mL H_2_O_2_ (30 wt%) was added to the system to initiate the catalytic reaction. At a predetermined time, 1 mL of solution was taken out for filtration, dilution, and then analysis. To investigate the effect of initial TC concentration on the removal of TC, a different concentrated TC solution (350–600 mg·L^−1^) was prepared. To study the effect of catalyst dosage, H_2_O_2,_ and HA, different numbers of Cu/C-SiO_2_ (0.1–1.0 g·L^−1^), H_2_O_2_ (5–15 mL), and HA (0.2735–0.6641 g) were added into the reaction solutions, respectively. pH of TC solution was adjusted from 3 to 11 by diluted HCl or NaOH solutions to study the effect of solution pH. To investigate the effect of water media, 500 mg·L^−1^ of TC solution was also prepared using tap water and pure water. In the trapping agent experiments, 40 mM (0.6486 g) of p-benzoquinone (BQ), or 10–200 mM (0.1152–2.3036 mL) of isopropyl alcohol (IPA), was added into the reaction solution with an initial TC concentration of 500 mg·L^−1^ and 15 mL of H_2_O_2_ in the system. In the cycling tests, the used Cu-C/SiO_2_ catalyst was calcined in N_2_ at 500 °C for 2 h for regeneration. 

The linear sweep voltammetry (LSV) curves and corresponding Tafel slopes were measured with 50 mM Na_2_SO_4_ as the supporting electrolyte on an electron-chemical workstation (CHI660D, Chenhua, Shanghai, China). The Cu/C-SiO_2_ catalyst was loaded on the glassy carbon electrode for detection. The powdered catalyst (5 mg) was firstly dispersed in ethanol (3 mL) by ultrasonic for 10 min to form a suspension. Next, 6.5 μL suspension and 1.7 μL Nafion solution (0.5 wt%) were poured onto the glassy carbon electrode. Then, the catalyst-loaded glassy carbon electrode was dried in a vacuum oven at 80 °C for detection. 

## 3. Results and Discussions

### 3.1. Structure and Composition of Cu/C-SiO_2_

**Figure 1:**
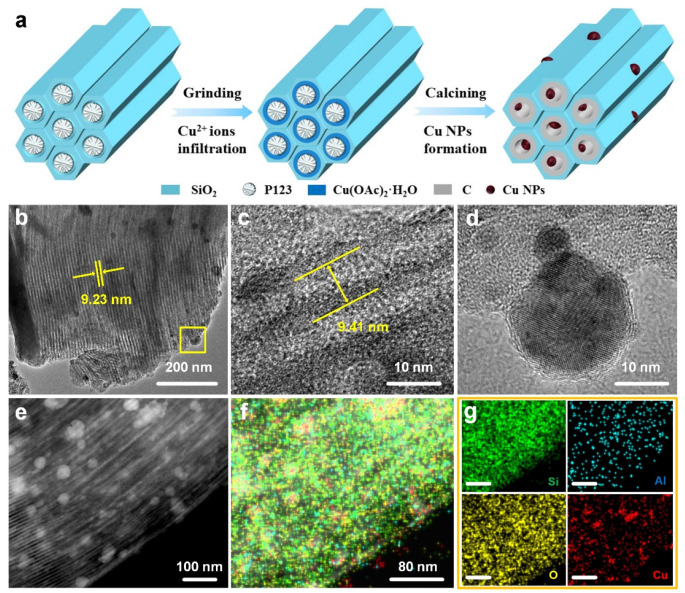
(**a**) Schematic illustration for the formation of Cu/C-SiO_2_. (**b**–**d**) TEM images of Cu/C-SiO_2_. (**e**) Dark-field STEM and (**f**,**g**) corresponding EDS element mapping images of Cu/C-SiO_2_. Scale bar in g: 80 nm).

Figure 1a shows the schematic diagram for the synthesis of Cu nanoparticles (NPs) incorporated mesoporous carbon-containing SiO_2_ (Cu/C-SiO_2_) through the grinding-assisted self-infiltration method. The P123-containing mesoporous SiO_2_ was firstly ground with the copper precursor (Cu(CH_3_COO)_2_·H_2_O) to obtain a homogeneous powder. Cupric ions (Cu^2+^) were spontaneously infiltrated into the confined space between the template (P123) and the siliceous walls driven by the shearing force of grinding [27,28]. Moreover, interactions between the Cu^2+^ and P123 happened in this process, since the color of the Cu(CH_3_COO)_2_·H_2_O + P123-containing SiO_2_ mixture changed from light blue to bluish-green after grinding. After the thermal treatment under N_2_, the P123 template was directly turned to carbon in the channels of mesoporous SiO_2_. At the same time, cupric ions were reduced to Cu NPs and incorporated into the carbon-containing mesoporous SiO_2_ (C-SiO_2_). The growth of Cu NPs was restricted in the mesopores and the matrix of C-SiO_2_ (Figure 1a).

TEM images indicate that Cu/C-SiO_2_ has uniform one-dimensional straight channels and Cu NPs are firmly embedded in the C-SiO_2_ matrix (Figure 1b). The average lattice parameter (*a*_0_) of the mesoporous Cu/C-SiO_2_ observed in Figure 1c is about 9.41 nm. Figure 1d shows a nanoparticle with obvious lattice fringes, which could be attributed to the crystal plane of Cu NPs [14]. The dark-field STEM image presents a selected area that has a high number of Cu NPs in the C-SiO_2_ matrix (Figure 1e). Corresponding EDS mapping images demonstrate that Si, O, and a small amount of Al are uniformly distributed on Cu/C-SiO_2_, and Cu is distributed on the support as a form of NPs (Figure 1g).

**Figure 2:**
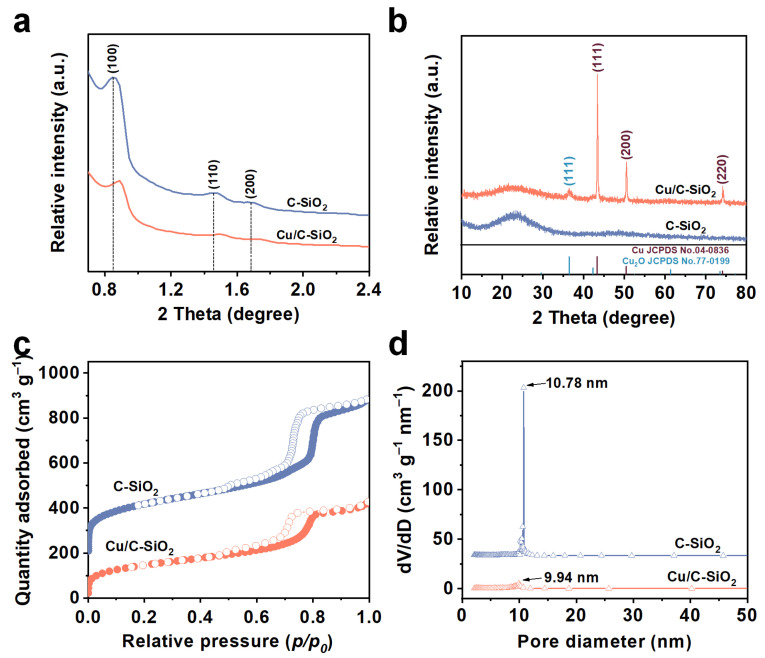
(**a**,**b**) Low- and wide-angle XRD patterns, (**c**,**d**) N_2_ adsorption–desorption isotherms, and pore size distribution (PSD) curves of C-SiO_2_ and Cu/C-SiO_2_. The isotherm and PSD curve for C-SiO_2_ was offset vertically by 200 cm^3^·g^–1^, and 33 cm^3^·g^–1^·nm^–1^ for clarification.

Figure 2a shows the low-angle XRD patterns of C-SiO_2_ and Cu/C-SiO_2_. Three diffraction peaks located at 0.87, 1.48, and 1.70° were observed for C-SiO_2_, which correspond to (100), (110), and (200) planes of the hexagonal space group, indicating the *p*6*mm* mesoporous structure [22]. The intensity of these three diffraction peaks declines, and the peaks shift to the higher angles (0.89, 1.52, and 1.75°, respectively) for Cu/C-SiO_2_, which is because Cu NPs partially occupied the mesopores the C-SiO_2_, leading to a decreased lattice parameter. Figure 2b presents the wide-angle XRD patterns of C-SiO_2_ and Cu/C-SiO_2_. The C-SiO_2_ shows a wide peak around 23°, typically identifying as an amorphous structure [29]. However, Cu/C-SiO_2_ displays three distinct XRD peaks at 43.3, 50.4, and 74.1°, respectively, which correspond to (111), (200), and (220) crystal planes of metallic Cu (JCPDS No. 04-0836) [14]. Moreover, a weak peak at 36.4° that corresponds to the (111) crystal plane of Cu_2_O is also observed for Cu/C-SiO_2_ [30], indicating the incomplete reduction of Cu^2+^ precursors during the calcination. Wide-angle XRD results indicate that Cu has two valences in Cu/C-SiO_2_, and metallic Cu is predominant while the content of univalent Cu is relatively low.

Figure 2c gives the N_2_ adsorption/desorption isotherms of C-SiO_2_ and Cu/C-SiO_2_. Both samples have type IV isotherms with typical H1-type hysteresis loops at high relative pressure (*p*/*p*_0_), revealing well-ordered mesopores in them [31,32]. Figure 2d shows that C-SiO_2_ has a very narrow pore size distribution (PSD) with a primary pore size of 10.78 nm. The PSD curve of the Cu NPs modified sample is a bit wider than that of C-SiO_2_, and the primary pore diameter is also smaller (9.94 nm), indicating partial blocking of the mesopores in Cu/C-SiO_2_. Moreover, textural properties display that the surface area and pore volume of Cu/C-SiO_2_ are 509 m^2^·g^–1^ and 0.65 cm^3^·g^–1^, respectively, which are smaller than those of C-SiO_2_ (756 m^2^·g^–1^ and 1.01 cm^3^·g^–1^) due to the presence of Cu NPs in the mesopores (Table S1). 

Figure S2 presents the FTIR spectra of C-SiO_2_, Cu/C-SiO_2_-AS, and Cu/C-SiO_2_. The absorption peaks at 3476 and 1632 cm^−1^ are attributed to the bending vibration (*δ*_OH_(H-O-H)) and telescopic vibration (*ν*_OH_(H-O-H)) in the water molecule, respectively [33]. The absorption peaks near 1076 and 802 cm^−1^ correspond to the asymmetric stretching vibrations of Si-O-Si [*ν*_as_(Si-O-Si)] and symmetrical stretching vibrations of Si-O-Si [*ν*_s_(Si-O-Si)] [34,35]. The characteristic peak at 960 cm^−1^ reflects the existence of independent Si-OH functional groups [36]. The small peak at 1387 cm^−1^ of C-SiO_2_ is attributed to the C=C stretching vibrations, which originated from the carbonized P123 [37]. The Cu/C-SiO_2_ composite appears with three extra peaks at 1445, 686, and 631 cm^−1^ different from the other two samples. The vibration of functional groups at 1445 cm^−1^ may be due to the delocalized C=C bonds [38], and the peak at 686 and 631 cm^−1^ are attributed to the presence of Cu-O-Si bonds and Cu_2_O in Cu/C-SiO_2_ [39,40].

**Figure 3:**
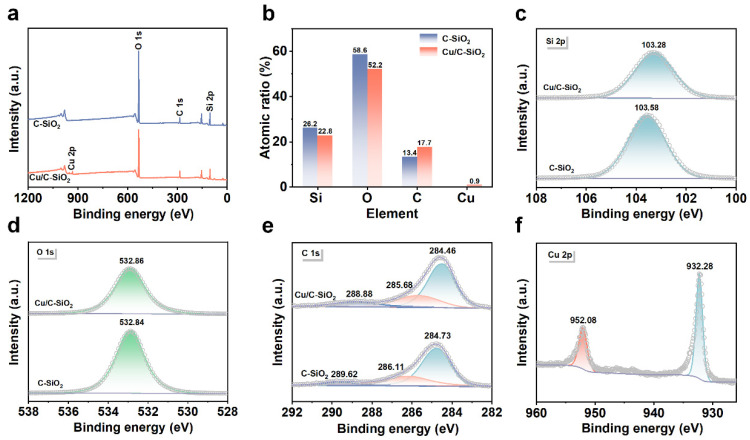
XPS results of C-SiO_2_ and Cu/C-SiO_2_. (**a**) Full scan, (**b**) surface atomic ratios, and high-resolution spectra of (**c**) Si 2p, (**d**) O 1s, (**e**) C 1s, and (**f**) Cu 2p.

XPS analysis was performed to further explore the chemical state of elements in C-SiO_2_ and Cu/C-SiO_2_. The characteristic peaks of O, Si, and C are observed in the XPS spectra of the two samples (Figure 3a). Surface contents of Si and O in Cu/C-SiO_2_ are slightly decreased if compared with C-SiO_2_ (Figure 3b). The Si 2p of C-SiO_2_ and Cu/C-SiO_2_ show peaks at binding energy (*B.E.*) of 103.58 and 103.28 eV, respectively, which belong to the Si-O-Si bond (Figure 3c) [41]. The blue shift of Cu/C-SiO_2_ may be induced by the interaction between Cu and Si. Both samples show an O1s XPS peak at *B.E.* of 532.84/532.86 eV, confirming the existence of Si-O-Si in them (Figure 3d) [42]. The C1s spectra of C-SiO_2_ can be divided into one primary peak at *B.E.* of 284.73 eV with two satellite peaks at 286.11 and 289.62 eV, which correspond to C-C, C-O-C, and O-C=O bond in the material (Figure 3e) [43,44]. These three peaks in Cu/C-SiO_2_ blue shift towards the lower *B.E.*, indicating the interactions between Cu and C. Moreover, Cu/C-SiO_2_ shows two XPS peaks at 933.28 and 952.08 eV, which are attributed to Cu 2p_3/2_ and Cu 2p_1/2_ of metallic Cu (Figure 3f) [45].

### 3.2. Catalytic Performance of Cu/C-SiO_2_

#### 3.2.1. Adsorption and Synergetic Catalytic Degradation of TC

**Figure 4:**
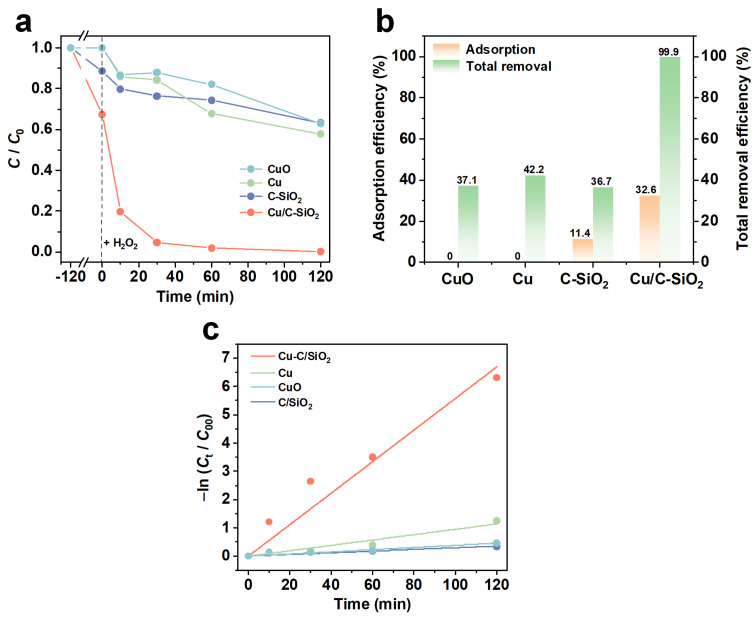
Adsorption and synergetic degradation of TC by CuO, Cu, C-SiO_2_, and Cu/C-SiO_2_: (**a**) *C*/*C*_0_ at different reaction times, (**b**) adsorption and total removal efficiencies in different systems, (**c**) fitted kinetic curves (catalyst dose = 1 g·L^−1^, *C*_0_ = 500 mg·L^−1^, pH = 3.65 ± 0.05, H_2_O_2_ = 15 mL).

Figure 4 shows the adsorption and synergistic degradation of TC (500 mg∙L^−1^) on different catalysts. The adsorption test was carried out for 2 h to preliminarily reach the adsorption equilibrium. In the adsorption process, commercial CuO and Cu have no adsorption capability towards TC since the removal efficiency is zero (Figure 4a,b). The TC adsorption efficiency by Cu/C-SiO_2_ is 32.6%, which is much higher than that of C-SiO_2_ (11.4%), indicating the incorporated Cu NPs are beneficial for improving adsorption towards TC. The improvement of adsorption efficiency towards TC over Cu/C-SiO_2_ is primarily attributed to the accessible active cupric sites, the porous structure, and the carbon in the mesoporous adsorbent/catalyst. It has been confirmed that the doped Cu species could remarkably increase the catalyst’s adsorption ability towards TC due to the complexation of TC and Cu species [46,47]. Moreover, the large surface area and pore volume of Cu/C-SiO_2_ also contribute to accommodating TC molecules inside its pores. In addition, the π-π interactions between the carbon and the ring structure of TC would also promote the adsorption property of Cu/C-SiO_2_ [48]. The improved adsorption is beneficial for enhancing the catalytic performance of the catalyst, and the adsorbed TC molecules can be rapidly degraded by those active radicals generated by the catalyst with the assistance of H_2_O_2_.

After adsorption for 2 h, 15 mL H_2_O_2_ was added to the system to initiate the catalytic reaction. In the system using C-SiO_2_, the concentration of TC was further decreased and the total removal efficiency (*R.E.*) reached 36.7% after further reaction for 2h. However, when using Cu/C-SiO_2_ as the catalyst, the highly concentrated TC solution (500 mg·L^−1^) was completely removed at a total reaction time of 4 h (*R.E.* = 99.9%). This demonstrates the remarkable catalytic activity of the Cu NPs that were incorporated in the mesoporous C-SiO_2_. For comparison, the catalytic activities of Cu and CuO were also determined. The total *R.E.* of Cu and CuO towards TC is 42.2% and 37.1%, respectively, further suggesting the active sites in Cu/C-SiO_2_ catalyst are related to cupric species. 

Figure 4c and Table S2 display the kinetic curves and reaction rate constants fitted by the pseudo-first-order kinetic model. The reaction rate coefficient (*k*) of Cu/C-SiO_2_ is 0.05576 min^−1^, which is at least five times larger than those of C-SiO_2_, Cu, and CuO (Table S2). This is possibly due to that the Cu NPs incorporated in mesoporous C-SiO_2_ have provided more accessible active sites for the decomposition of TC, and the meso-channels of SiO_2_ are beneficial for improving the mass transfer of molecules as well. Table S3 lists the removal capacities of different catalysts reported in the literature towards TC [49,50,51,52,53]. The Cu/C-SiO_2_ material has a high removal ability of TC.

#### 3.2.2. Effect of Initial TC Concentration

**Figure 5:**
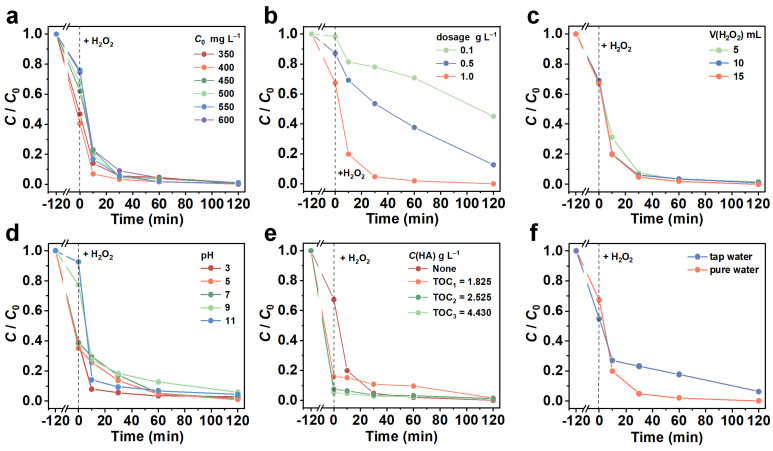
Effects of (**a**) initial TC concentration, (**b**) catalyst dosage, (**c**) H_2_O_2_ dosage, (**d**) pH, (**e**) HA, and (**f**) water media on TC degradation.

Figure 5a and Figure S3a show the adsorption and synergistic degradation of TC with different initial concentrations (350–600 mg·L^−1^) by Cu/C-SiO_2_. The results show that the TC adsorption efficiency by Cu/C-SiO_2_ was slightly increased from 53.2% to 59.5% first, then decreased to 38.0%, 32.6%, 24.0%, and 25.4%, respectively, when the initial TC concentration was raised from 350 mg·L^−1^ to 600 mg·L^−1^. After the introduction of H_2_O_2_, catalytic degradation of TC happened, and the total *R.E.* towards TC in all highly concentrated TC systems (350–600 mg·L^−1^) is larger than 99% by using Cu/C-SiO_2_ as the catalyst (Figure 5a and Figure S3a). The fitted rate coefficient is in the range of 0.03917–0.05576 min^−1^, demonstrating the excellent catalytic performance of Cu/C-SiO_2_ (Figure S3b). 

#### 3.2.3. Effect of Catalyst Dosage

Figure 5b and Figure S4a show the removal of TC (*C*_0_ = 500 mg·L^−1^) with different catalyst dosages. The TC adsorption efficiency is about 2%, and the total *R.E.* is 55% when the catalyst dosage is 0.1 g·L^−1^. When the catalyst dosage is increased to 0.5 g·L^−1^, the *R.E.* by adsorption is 13%, and the total *R.E.* reaches 87%. Further raising the catalyst dosage to 1.0 g·L^−1^, the total *R.E* achieves 99.9% after the reaction for 4 h. Moreover, the degradation rate constant (*k*) also increases with the increase in the catalyst dosage (Figure S4b), indicating that the increased dosage of Cu/C-SiO_2_ is beneficial for both improving the degradation capability and the rate towards TC in this catalytic system. 

#### 3.2.4. Effect of H_2_O_2_ Dosage

Figure 5c and Figure S5 show the effect of H_2_O_2_ dosage on the removal of TC. The total *R.E.* under reaction for 4 h is 98.4, 99.0, and 99.9% for the system adding 5, 10, and 15 mL of H_2_O_2_, demonstrating a positive tendency of TC degradation with the increase in H_2_O_2_ addition. It is found that introducing 10 mL of H_2_O_2_ is enough to initiate and almost completely remove the highly concentrated TC (*C*_0_ = 500 mg·L^−1^). Moreover, the *k* values for these three systems are 0.03721, 0.04088, and 0.05576 min^−1^, respectively (Figure S5b), suggesting a faster reaction rate for the system with the higher number of H_2_O_2_ addition.

#### 3.2.5. Effect of Solution pH

Figure 5d shows the effect of solution pH on the adsorption and degradation of TC. When the solution pH increases from 3 to 7, the amount of adsorbed TC by Cu/C-SiO_2_ is relatively large (*R.E.* = 61.1–64.9%) (Figure 5d and Figure S6a). This is due to that the surface of Cu/C-SiO_2_ is negatively charged in the pH range of 3–11, while the TC is positively charged or electrically neutral when pH < 7.7 (Figure S6c,d). Electrostatic interaction between Cu/C-SiO_2_ and TC plays a primary role in the adsorption under pH < 7.7. When pH is greater than 7.7, the adsorption capacity decreases with the increase in solution pH, which is attributed to the electrostatic repulsion between Cu/C-SiO_2_ and TC since TC is negatively charged when pH > 7.7 (Figure S6d) [54]. Solution pH has a relatively small effect on the degradation of TC, and the total *R.E.* is in the range of 94–99% at pH = 3.0–11.0 (Figure 5d and Figure S6a), indicating that Cu/C-SiO_2_ can be applied in a wide pH range.

#### 3.2.6. Effect of HA

Humic acid (HA) is a kind of macromolecular organic matter that widely exists in the environment. Figure 5e displays the adsorption and synergistic degradation of TC by Cu/C-SiO_2_ in the reaction solution by adding different amounts of HA to it. The concentration of HA was expressed as total organic carbon (TOC). Results show that the addition of HA in the reaction solution greatly boosts the adsorption of TC. The adsorption efficiency towards TC increases from 32.6% to 84.0%, 92.5%, and 94.9%, respectively, along with the HA concentration raises from 0 to TOC of HA = 1.825, 2.525, and 4.430 mg·L^−1^. This is probably due to that functional groups such as -COOH and -OH in HA would combine with TC molecules by hydrogen bonding and other forces, and then promote the removal of TC [55]. After the introduction of H_2_O_2_ into the system, the final *R.E.* of these systems containing different amounts of HA (TOC = 1.825, 2.525, and 4.430 mg·L^−1^) also reaches 98.4%, 98.7%, and 99.0%, respectively. This result indicates that adsorption plays a very important role in the removal of TC in the system containing HA.

#### 3.2.7. Effect of Water Media

Figure 5f demonstrates the removal of TC by Cu/C-SiO_2_ in different aqueous media. The Cu/C-SiO_2_ shows an adsorption efficiency of 45.0% towards TC in a tap water medium, which is a bit larger than that in a pure water medium. This implies that impurity ions in tap water may have a positive effect on the adsorption of TC by Cu/C-SiO_2_. However, the total *R.E.* of TC in tap water is 94.0%, which is lower than that in pure water (99.9%), indicating that the catalytic ability of Cu/C-SiO_2_ is affected by the water quality.

### 3.3. Proposed Mechanism for TC Degradation over Cu/C-SiO_2_

**Figure 6:**
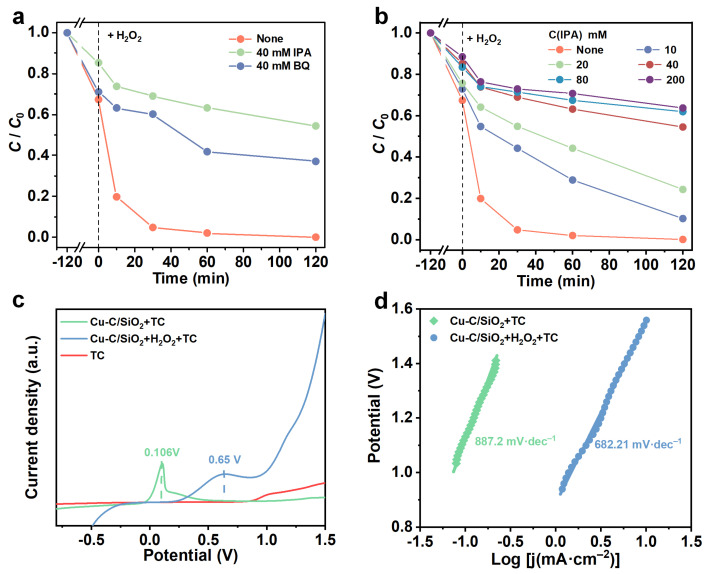
(**a**) *C*/*C*_0_ at different reaction times with the addition of different quenching scavengers in the Cu/C-SiO_2_+H_2_O_2_ system, and (**b**) *C*/*C*_0_ at different reaction times with the addition of different IPA in the Cu/C-SiO_2_+H_2_O_2_ system. (**c**) LSV curves, and (**d**) Tafel slopes obtained using Cu/C-SiO_2_ as electrode material in different reaction conditions.

Scavenging experiments were carried out to investigate the primary reactive species during the TC degradation process by Cu/C-SiO_2_ (Figure 6a and Figure S8). BQ and IPA were applied as scavengers of the superoxide free radicals (•O_2_^−^), and hydroxyl free radicals (•OH), respectively [56,57]. The removal percentage of TC decreased from 99.9% to 62.8% when an extra 40 mM of BQ was added into the system, implying the contribution of •O_2_^−^ in this reaction system. Meanwhile, the *R.E.* of TC dramatically declines from 99.9% to 45.5% after the addition of 40 mM IPA into the system. This indicates that •OH contributes more than •O_2_^−^ in this TC-Cu/C-SiO_2_-H_2_O_2_ catalytic system. 

A different number of IPA was introduced into the reaction system to further investigate the function of •OH during the catalytic process. The total *R.E.* of TC decreased gradually with the increase in IPA addition (Figure 6b and Figure S9). When the number of added IPA is relatively low (10 and 20 mM), the *R.E.* of TC decreased to 89.8% and 75.7%. When the introduced IPA number is high (100 and 200 mM), the *R.E.* of TC became 38.1 and 36.3%. This phenomenon confirms that •OH plays a dominant role in this TC-Cu/C-SiO_2_-H_2_O_2_ catalytic system. Besides, •O_2_^−^ also contributes to the degradation of TC over Cu/C-SiO_2_. 

Moreover, we have also performed linear sweep voltammetry (LSV) to measure the redox processes between the catalyst and TC molecules with or without H_2_O_2_. The LSV curves in Figure 6c indicate that no visible oxidation peaks appear in the TC solution. However, Cu/C-SiO_2_ has an oxidation peak at ca. 0.11V in TC solution, indicating redox reactions happened between Cu/C-SiO_2_ and TC. Comparatively, Cu/C-SiO_2_ presents a much larger oxidation peak at a higher potential (0.65V) in the system containing both TC and H_2_O_2_, implying the enhanced redox ability of Cu/C-SiO_2_ with the assistance of H_2_O_2_. Furthermore, Cu/C-SiO_2_ also exhibits a lower Tafel slope in the TC+H_2_O_2_ reaction system than in pure TC solution, suggesting faster reaction dynamics after the addition of H_2_O_2_ into the reaction solution (Figure 6d). 

It was reported that the multivalent Cu species present redox ability similar to traditional Fe Fenton catalysts, and H_2_O_2_ can be decomposed into •OH and •O_2_^−^ by Cu oxides [58]. Based on the above experimental results, it is suggested that TC decomposition in this Cu/C-SiO_2_-H_2_O_2_ system may involve Equations (1)–(8). The following steps are suggested: (i) TC molecules adsorbed onto Cu/C-SiO_2_ through complex interactions, including electrostatic and coordination interactions [54]. (ii) The multivalent Cu species in Cu/C-SiO_2_ reacted with H_2_O_2_ and accelerated the formation of •OH and •O_2_^−^ free radicals (Equations (1)–(6)). (iii) TC molecules were attached and simultaneously degraded by those active free radicals (Equations (7) and (8)). We also observed that the pH value of the reaction solution was increased from 3.65 to 4.30 along with the reaction time, demonstrating the generation of OH^−^ during the catalytic process. Moreover, the pH of the reaction solution that was added with IPA increased only to 3.85, indicating that the addition of IPA strongly inhibits the formation of •OH in the system.

(1) $${Cu}^{0}+H_{2}O_{2}\to {Cu}^{+}+∙\mathrm{OH}+{OH}^{-}\;(E^{\theta }=2.280\;V)$$

(2) $${Cu}^{+}+H_{2}O_{2}\to {Cu}^{2+}+∙\mathrm{OH}+{OH}^{-}\;(E^{\theta }=2.641\;V)$$

(3) $${2Cu}^{0}+O_{2}+H_{2}O\to {2Cu}^{2+}+4{OH}^{-}\;(E^{\theta }=0.060\;V)$$

(4) $${Cu}^{2+}+H_{2}O_{2}+{OH}^{-}\to {Cu}^{+}+{∙HO}_{2}+H_{2}O(E^{\theta }=1.351\;V)$$

(5) $${Cu}^{+}+H_{2}O_{2}+{OH}^{-}\to {Cu}^{0}+{∙HO}_{2}+H_{2}O(E^{\theta }=0.990\;V)$$

(6) $${∙HO}_{2}+H_{2}O\to ∙O_{2}^{-}+H_{3}^{+}O$$

(7) $$∙O_{2}^{-}+\mathrm{TC}\to \;\mathrm{intermediates}\to {CO}_{2}+H_{2}O$$

(8) $$∙\mathrm{OH}+\mathrm{TC}\to \;\mathrm{intermediates}\to {CO}_{2}+H_{2}O$$

**Figure 7:**
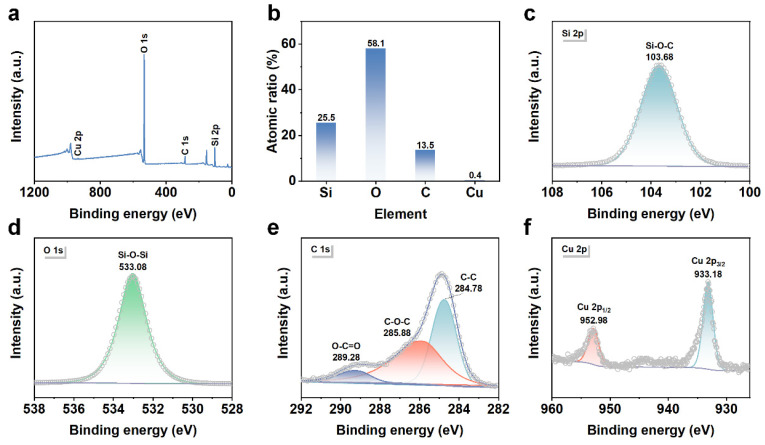
XPS patterns of Cu/C-SiO_2_ after reaction: (**a**) full spectra, (**b**) atomic ratios, (**c**) Si 2p, (**d**) O 1s, (**e**) C 1s, and (**f**) Cu 2p.

To further explore the reaction mechanism of catalytic degradation of TC by Cu/C-SiO_2_, the surface chemical states of elements in Cu/C-SiO_2_ after the reaction were studied by XPS (Figure 7). Characteristic peaks of Si, O, C, and Cu are observed in the reacted Cu/C-SiO_2_ (Figure 7a). Moreover, the surface content of Cu was decreased from 0.9 to 0.4 after the catalytic reaction, which is probably due to the metal leaching during the Fenton-like catalytic reaction (Figure 3b and Figure 7b). XPS spectrum of Si 2p shows a bit higher *B.E.* (103.68 eV) corresponds to Si-O-C and the *B.E.* of O 1s also shifts to a relatively high value (533.08 eV) after the reaction (Figure 3c,d and Figure 7c,d) [42]. Moreover, the reacted Cu/C-SiO_2_ also displays C 1s peaks at 284.78, 285.88, and 289.28 eV, which correspond to the C-C, C-O-C, and O-C=O bonds, respectively (Figure 7e) [43,44]. The *B.E.* values of these C 1s peaks for Cu/C-SiO_2_ after the reaction are also higher than those for the sample before the reaction (Figure 3e and Figure 7e). This indicates that the support C-SiO_2_ might be involved in the reaction. The Cu 2p peaks of Cu/C-SiO_2_ after the reaction also show a positive *B.E.* shift of about 0.90 eV if compared with those of the sample before the reaction (Figure 3f and Figure 7f). The *B.E.* values of Cu 2p_3/2_ and Cu 2p_1/2_ are centered at 933.18 and 952.98 eV, respectively, which implies monovalent copper (Cu^+^) is the dominant species in the reacted Cu/C-SiO_2_ catalyst [45]. In addition, an inconspicuous shake-up satellite between 935 and 950 eV can be observed in Figure 7f, implying the presence of a small number of divalent copper (Cu^2+^) species in the sample after the reaction [59].

### 3.4. Reusability of the Cu/C-SiO_2_

Figure S10 shows the cycling performance of Cu-C/SiO_2_ for the adsorption and degradation of TC (500 mg·L^−1^). In the first run, the total *R.E.* towards TC reaches 99% with a rapid degradation rate. In the second cycle, the *R.E.* towards TC remains at 98% with a slower degradation rate. In addition, the TC adsorption efficiency is reduced from 46% to 18% (Figure S10, blue line). This may be due to the leaching of Cu species from the Cu-C/SiO_2_ catalyst since 37.5 mg·L^−1^ of Cu was detected from the reaction solution after the first run. However, the leaching amount of Cu decreased to 4.9 mg·L^−1^ after the second cycle, indicating relative stability of Cu-C/SiO_2_. In the third cycle, the TC adsorption efficiency is about 12%, and the total *R.E.* declines to 58%, implying the decreased catalytic activity of Cu-C/SiO_2_. In this cycle, only 1.12 mg·L^−1^ of Cu was detected in the reacted solution, suggesting that Cu-C/SiO_2_ is relatively stable after 3 recycled catalytic reactions.

## 4. Conclusions

In summary, we developed novel Cu NPs-doped mesoporous C-SiO_2_ composite through pre-infiltrating Cu precursors into the P123-containing mesoporous SiO_2_, followed by a synchronously reducing Cu and C precursors in the mesopores of SiO_2_. The Cu/C-SiO_2_ maintains the structural ordering of the mesoporous SiO_2_, and Cu NPs are effectively immobilized in the matrix of C-SiO_2_ since the growth of Cu NPs was restricted by C and the confined space in C-SiO_2_. The synthesized Cu/C-SiO_2_ was then served as a heterogeneous Fenton-like catalyst and it presents excellent catalytic properties. It can remove 99.9% of the highly concentrated (*C*_0_ = 500 mg·L^−1^) within a total reaction time of 4h. Moreover, this catalyst also shows good pH TC adaptability, and the removal efficiency towards TC is 94–99% at pH = 3.0–11.0. The work provides a new feasible strategy for the synthesis and application of Cu-based heterogeneous catalysts for highly efficient treatment of antibiotics wastewater.

## Data Availability

Data will be available on request.

## Supplementary Materials

The following supporting information can be downloaded at: https://www.mdpi.com/article/10.3390/nano13172478/s1, Text S1: Characterization of the catalysts; Text S2: Analytical methods for adsorption and catalysis; Table S1: N_2_ adsorption/desorption results of the C-SiO_2_ and Cu/C-SiO_2_; Table S2: Reaction rate constants of TC degradation by CuO, Cu, C-SiO_2_, and Cu/C-SiO_2_; Table S3: The catalytic capacity of different catalysts towards TC; Figure S1: TG and DSC curves of the template-containing mesoporous SiO_2_ samples; Figure S2: FTIR spectra of C-SiO_2_, Cu/C-SiO_2_-AS and Cu/C-SiO_2_; Figure S3: Effect of initial TC concentration on the removal of TC (catalyst dosage = 1 g·L^−1^, *C*_0_ = 350–600 mg·L^−1^, H_2_O_2_ = 15 mL, T = 20 ± 2 °C): (a) adsorption and total removal efficiencies, (b) fitted rate constant *k*; Figure S4: Effect of catalyst dosage on the removal of TC: (a) adsorption and total removal efficiencies, (b) fitted rate constant *k* (*C*_0_ = 500 mg·L^−1^, H_2_O_2_ = 15 mL); Figure S5: Effect of H_2_O_2_ dosage on the removal of TC: (a) adsorption and total removal efficiencies, (b) fitted rate constant *k* (catalyst dosage = 1 g·L^−1^, *C*_0_ = 500 mg·L^−1^); Figure S6: Effect of pH on the removal of TC: (a) adsorption and total removal efficiencies, (b) fitted rate constant k, (c) Zeta potentials of Cu/C-SiO_2_ at different pH values and (d) the molecular formula for TC (catalyst dose = 1 g·L^−1^, C_0_ = 500 mg·L^−1^, pH = 3.65 ± 0.05, H_2_O_2_ = 15 mL); Figure S7: Effect of HA on the removal of TC: (a) adsorption and total removal efficiencies, (b) fitted rate constant *k* (catalyst dosage = 1 g·L^−1^, *C*_0_ = 500 mg·L^−1^, pH = 3.65 ± 0.05, H_2_O_2_ = 15 mL); Figure S8: Different quenching scavengers in the Cu/C-SiO_2_ + TC system: (a) adsorption and total removal efficiencies, (b) fitted rate constant k (catalyst dosage = 1 g·L^−1^, *C*_0_ = 500 mg·L^−1^, pH = 3.65 ± 0.05, H_2_O_2_ = 15 mL); Figure S9: Different concentrations of IPA in the Cu/C-SiO_2_ + TC system: (a) adsorption and total removal efficiencies, (b) fitted rate constant k (catalyst dosage = 1 g·L^−1^, *C*_0_ = 500 mg·L^−1^, pH = 3.65 ± 0.05, H_2_O_2_ = 15 mL); Figure S10: Cyclic performance of Cu/C-SiO_2_ + TC system: (a) adsorption and total removal efficiencies, (b) fitted rate constant *k* (catalyst dosage = 1 g·L^−1^, *C*_0_ = 500 mg·L^−1^, pH = 3.65 ± 0.05, H_2_O_2_ = 15 mL). References [49,50,51,52,53] are cited in the supplementary materials.
