# Supplementary material for: Synthesis of Cu Nanoparticles Incorporated Mesoporous C/SiO2 for Efficient Tetracycline Degradation
Source: Nanomaterials (Basel). 2023 Sep 2;13(17):2478. doi: 10.3390/nano13172478 (PMC10489891; doi:10.3390/nano13172478)
Supplement: Supplementary file 1 [file nanomaterials-13-02478-s001.zip › nanomaterials-2553314-supplementary.pdf]

Synthesis of Cu Nanoparticles Incorporated Mesoporous C/SiO_2 for Efficient Tetracycline Degradation

Ning Wang[1,2] Yuanyuan Zhao,[1,2] Xuelian Wu,[1,2] Dapeng Li,[3] Ruguang Ma,[1] Zhigang Chen,[1,3] and Zhengying Wu,[1,3]*

[1] Jiangsu Key Laboratory for Environment Functional Materials, School of Materials Science and Engineering, Suzhou University of Science and Technology, Suzhou 215009, China

[2] School of Chemistry and Life Science, Suzhou University of Science and Technology, Suzhou 215009, China

[3] Jiangsu Collaborative Innovation Center of Technology and Material for Water Treatment, Suzhou University of Science and Technology, Suzhou 215009, China

**Corresponding authors:*

Zhengying Wu, Tel./fax: +86 512-67374120, Email: zywu@mail.usts.edu.cn;

Supporting information Texts..S4

Text S1. Characterization of the catalysts 4

Text S2. Analytical methods for adsorption and catalysis....................................5

Supporting information Table..S6

Table S1. N_2 adsorption/desorption results of the C-SiO_2 and Cu/C-SiO_2. 6

Table S2. Reaction rate constants of TC degradation by CuO, Cu, C-SiO_2, and Cu/C-SiO_2.. 7

Table S3. The catalytic capacity of different catalysts towards TC. 8

Supporting information Figures..S9

Figure S1. TG and DSC curves of the template-containing mesoporous SiO_2 sample. 9

Figure S2. FTIR spectra of C-SiO_2, Cu/C-SiO_2-AS, and Cu/C-SiO_2. 10

Figure S3. Effect of initial TC concentration on the removal of TC (catalyst dosage = 1 g·L^{-1}, C_0 = 350−600 mg·L^{-1}, H_2O_2 = 15 mL, T = 20±2 °C): (a) adsorption and total removal efficiencies, (b) fitted rate constant *k*..11

Figure S4. Effect of catalyst dosage on the removal of TC: (a) adsorption and total removal efficiencies, (b) fitted rate constant *k* (C_0 = 500 mg·L^{-1}, H_2O_2 = 15 mL). 12

Figure S5. Effect of H_2O_2 dosage on the removal of TC: (a) adsorption and total removal efficiencies, (b) fitted rate constant *k* (catalyst dosage = 1 g·L^{-1}, C_0 = 500 mg·L^{-1}) 13

Figure S6. Effect of pH on the removal of TC: (a) adsorption and total removal

efficiencies, (b) fitted rate constant k, (c) Zeta potentials of Cu/C-SiO_2 at different pH values and (d) the molecular formula for TC...14

Figure S7. Effect of HA on the removal of TC: (a) adsorption and total removal efficiencies, (b) fitted rate constant k (catalyst dosage = 1 g·L^{-1}, C_0 = 500 mg·L^{-1}, pH = 3.65 ± 0.05, H_2O_2 = 15 mL)..15

Figure S8. Different quenching scavengers in the Cu/C-SiO_2 + TC system: (a) adsorption and total removal efficiencies, (b) fitted rate constant k (catalyst dosage = 1 g·L^{-1}, C_0 = 500 mg·L^{-1}, pH = 3.65 ± 0.05, H_2O_2 = 15 mL)...........................16

Figure S9. Different concentrations of IPA in the Cu/C-SiO_2 + TC system: (a) adsorption and total removal efficiencies, (b) fitted rate constant k (catalyst dosage = 1 g·L^{-1}, C_0 = 500 mg·L^{-1}, pH = 3.65 ± 0.05, H_2O_2 = 15 mL)...........................17

Figure S10. Cyclic performance of Cu/C-SiO_2 + TC system: (a) adsorption and total removal efficiencies, (b) fitted rate constant k (catalyst dosage = 1 g·L^{-1}, C_0 = 500 mg·L^{-1}, pH = 3.65 ± 0.05, H_2O_2 = 15 mL). 18

Supporting information Texts

Text S1. Characterization of the catalysts

The synthesized Cu/C-SiO_2 was characterized by transmission electron microscopy (TEM, JEOL 2100F) with an energy-dispersive X-ray spectrometer (EDS, Bruker XFlash 6T|60) elemental mapping. The mesoporous structure and phase components of the material were determined using an X-ray diffractometer (XRD, Bruker D8 Advance, Cu Kα radiation, Germany). The N_2 adsorption-desorption isotherms were measured by a Micromeritics TriStar II 3020 system. The specific surface area and the pore size distribution of the material were calculated by the (BET) and Barrett-Joyner-Halenda (BJH) methods. Thermogravimetric (TG) analysis was conducted using a Perkin-Elmer instrument (SDT Q-600) with a heating rate of 20 °C min^{-1} up to 800 °C under airflow. Surface groups and chemical states of the elements for the materials were examined by Fourier transform infrared (FTIR) spectra (Thermo Nicolet IS 10), and X-ray photoelectron spectroscopy (XPS, Thermo Fisher Scientific ESCALAB 250) measurements. Zeta potentials of the Cu/C-SiO_2 at different initial pH values were measured by Zeta-sizer Nano-ZS (Malvern ZEN3690). The TC concentration was determined by using a TU-1901 spectrophotometer at a maximum wavelength of around 357 nm.

Text S2. Analytical methods for adsorption and catalysis

The samples were taken by disposable syringe at different times and then filtrate was immediately collected through 0.45 μm filter membrane to measure the absorbance. The catalytic degradation curve was fitted by the first-order model (Eq. (1)):

$$-\ln(C_0/C_t) = kt \tag{1}$$

Among them, C_0 and C_t are the initial concentration of TC and the concentration at time t, $mg \cdot L^{-1}$; k is the reaction rate coefficient, min^{-1}; t is the reaction time, h.

Supporting information Table

Table S1. N_2 adsorption/desorption results of the C-SiO_2 and Cu/C-SiO_2.

Sample	S_{BET} ($m^2 \cdot g^{-1}$)	V_P ($cm^3 \cdot g^{-1}$)	D_{BJH} (nm)
C/SiO_2	756	1.01	10.78
Cu-C/SiO_2(0.2)	509	0.65	9.94

D_{BJH}: pore diameters were calculated from the adsorption branches.

Table S2. Reaction rate constants of TC degradation by CuO, Cu, C-SiO_2, and Cu/C-SiO_2.

Sample	reaction rate constant (k, min^{-1})
Cu-C/SiO_2	0.05576
Cu	0.00957
CuO	0.00383
C/SiO_2	0.00296

Table S3. The catalytic capacity of different catalyst towards TC.

catalyst	time (h)	C_0 (mg·L^{-1})	dose (g·L^{-1})	C/C_0 (%)	Reference
$CuFeO_2$/BC	300	20	0.6	89.1	[1]
$CuFeO_2$-NO/PBC	180	20	0.8	96.1	[2]
Cu/$CuFe_2O_4$	1120	50	0.3	75.4	[3]
SAS-Cu	30	20	0.1	82.5	[4]
$Fe_{0.25}Cu_{0.75}$(BDC)@DE	120	20	0.5	93.0	[5]
Cu/C-SiO_2	240	500	1.0	99.9	This work

Supporting information Figures

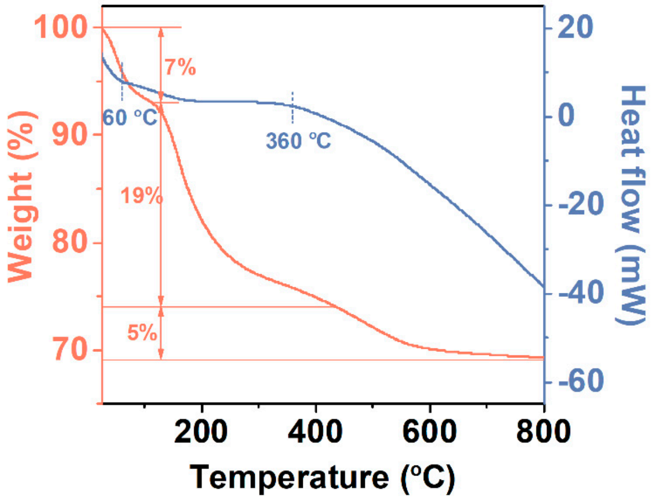

Figure S1. TG and DSC curves of the template-containing mesoporous SiO_2 sample.

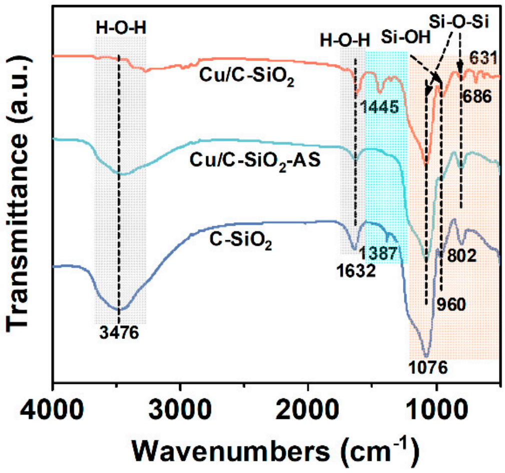

Figure S2. FTIR spectra of C-SiO_2, Cu/C-SiO_2-AS, and Cu/C-SiO_2.

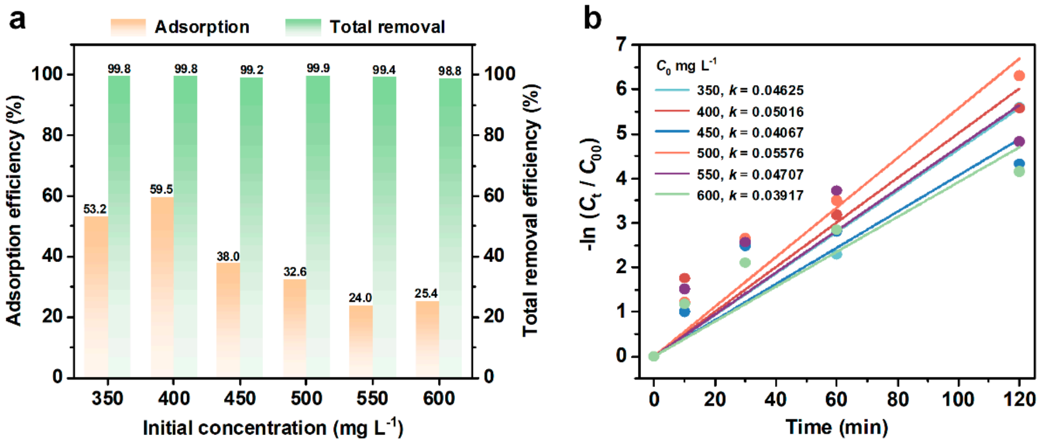

Figure S3. Effect of initial TC concentration on the removal of TC (catalyst dosage = 1 g·L^{-1}, C_0 = 350−600 mg·L^{-1}, H_2O_2 = 15 mL, T = 20±2 °C): (a) adsorption and total removal efficiencies, (b) fitted rate constant *k*.

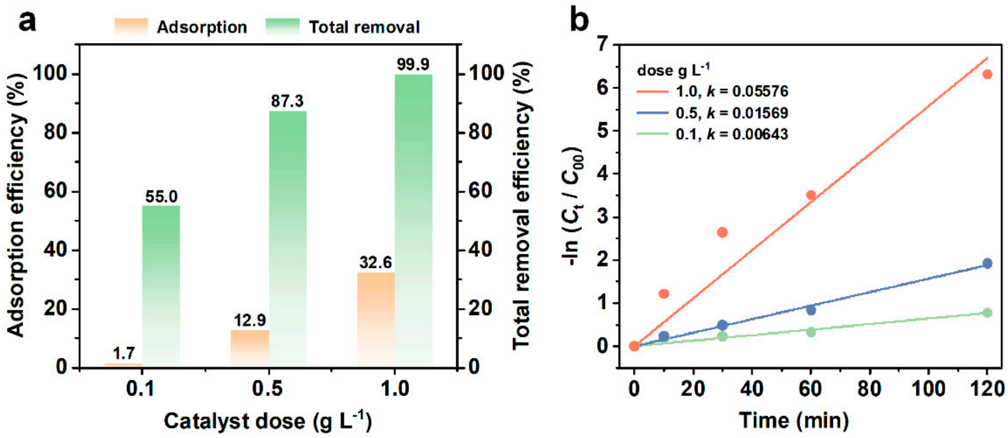

Figure S4. Effect of catalyst dosage on the removal of TC: (a) adsorption and total removal efficiencies, (b) fitted rate constant *k* (C_0 = 500 mg·L^{-1}, H_2O_2 = 15 mL).

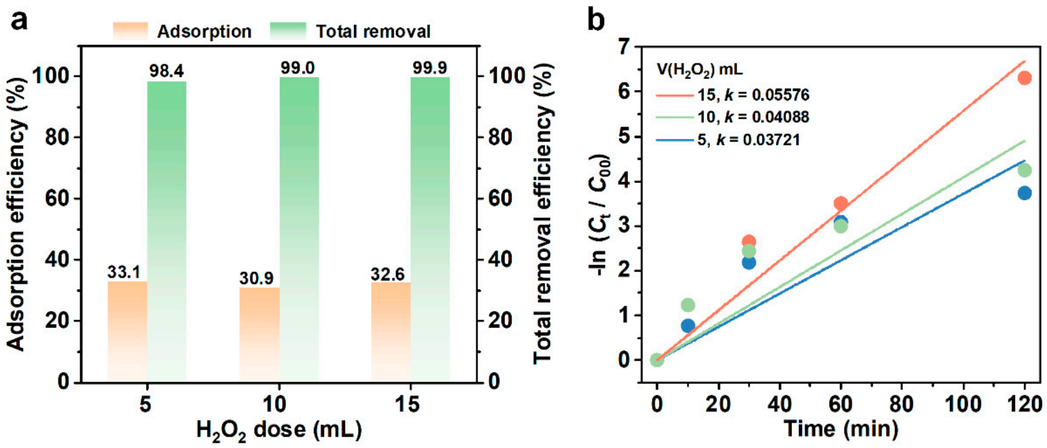

Figure S5. Effect of H_2O_2 dosage on the removal of TC: (a) adsorption and total removal efficiencies, (b) fitted rate constant k (catalyst dosage = 1 g·L^{-1}, C_0 = 500 mg·L^{-1})

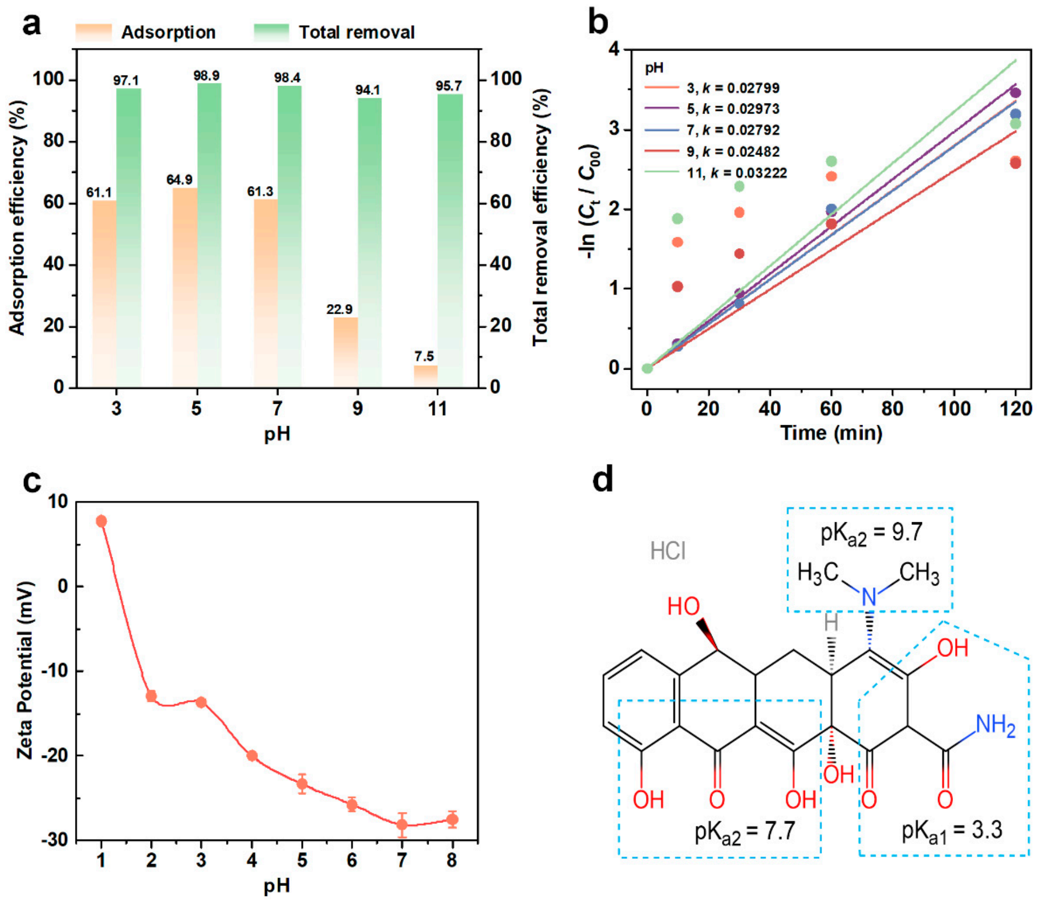

Figure S6. Effect of pH on the removal of TC: (a) adsorption and total removal efficiencies, (b) fitted rate constant *k*, (c) Zeta potentials of Cu/C-SiO_2 at different pH values and (d) the molecular formula for TC.

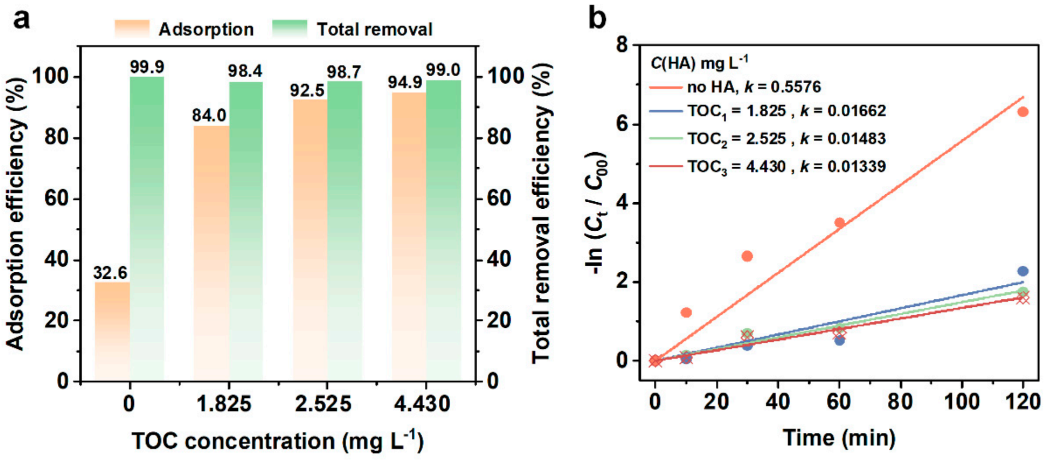

Figure S7. Effect of HA on the removal of TC: (a) adsorption and total removal efficiencies, (b) fitted rate constant k (catalyst dosage = 1 g·L^{-1}, C_0 = 500 mg·L^{-1}, pH = 3.65 ± 0.05, H_2O_2 = 15 mL).

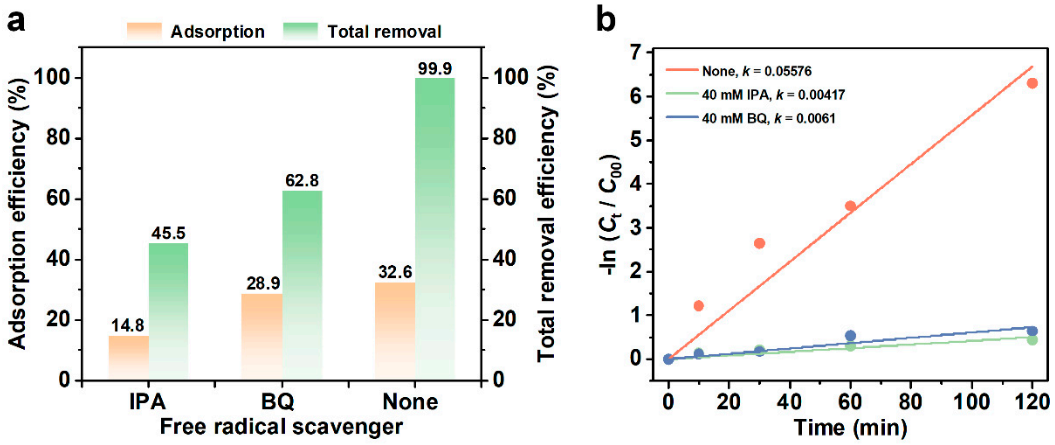

Figure S8. Different quenching scavengers in the Cu/C-SiO_2 + TC system: (a) adsorption and total removal efficiencies, (b) fitted rate constant *k* (catalyst dosage = 1 g·L^{-1}, C_0 = 500 mg·L^{-1}, pH = 3.65 ± 0.05, H_2O_2 = 15 mL).

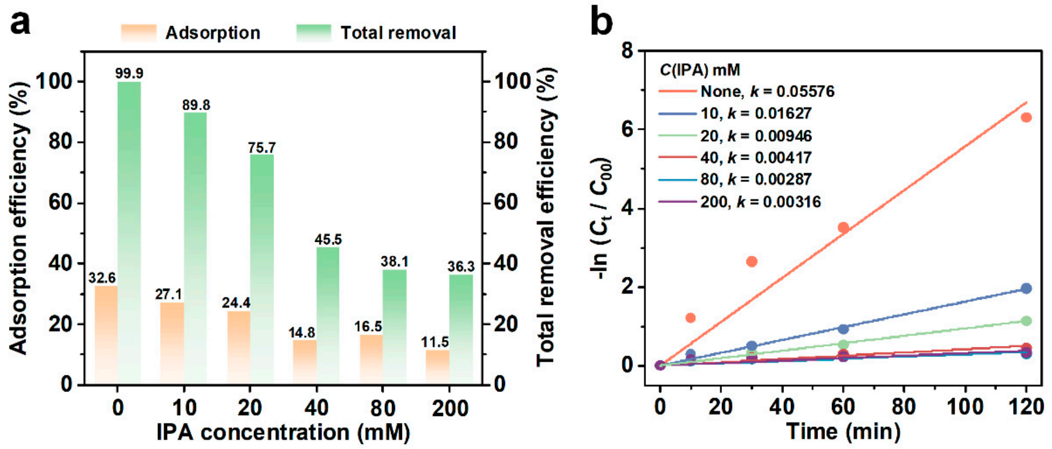

Figure S9. Different concentrations of IPA in the Cu/C-SiO_2 + TC system: (a) adsorption and total removal efficiencies, (b) fitted rate constant *k* (catalyst dosage = 1 g·L^{-1}, C_0 = 500 mg·L^{-1}, pH = 3.65 ± 0.05, H_2O_2 = 15 mL).

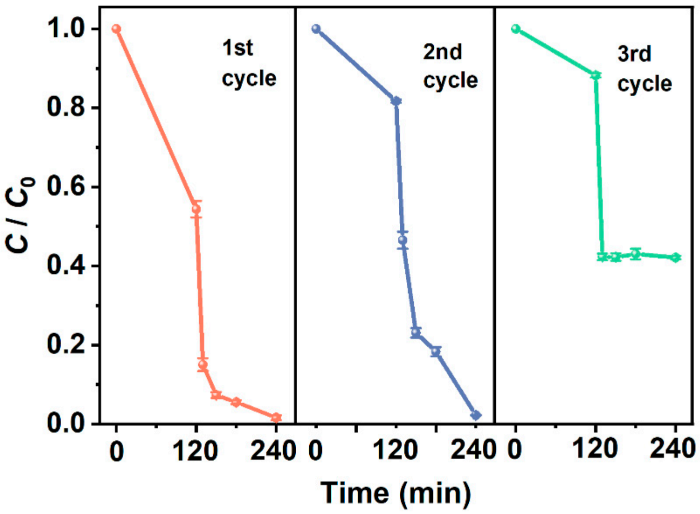

Figure S10. Cycling performance of Cu/C-SiO_2 (Adsorption for 2 hours, and catalytic reaction for 2 hours. Catalyst dosage = 1 g·L^{-1}, C_0 = 500 mg·L^{-1}, pH = 3.65 ± 0.05, H_2O_2 = 15 mL).

Reference

1. Xin, S.; Liu, G.; Ma, X.; Gong, J.; Ma, B.; Yan, Q.; Chen, Q.; Ma, D.; Zhang, G.; Gao, M.; et al. High efficiency heterogeneous Fenton-like catalyst biochar modified $CuFeO_2$ for the degradation of tetracycline: Economical synthesis, catalytic performance and mechanism. Applied Catalysis B: Environmental 2021, 280, 119386, doi:10.1016/j.apcatb.2020.119386.
2. Xin, S.; Huo, S.; Xin, Y.; Gao, M.; Wang, Y.; Liu, W.; Zhang, C.; Ma, X. Heterogeneous photo-electro-Fenton degradation of tetracycline through nitrogen/oxygen self-doped porous biochar supported $CuFeO_2$ multifunctional cathode catalyst under visible light. Applied Catalysis B: Environmental 2022, 312, 121442, doi:10.1016/j.apcatb.2022.121442.
3. Li, Z.; Guo, C.; Lyu, J.; Hu, Z.; Ge, M. Tetracycline degradation by persulfate activated with magnetic $Cu/CuFe_2O_4$ composite: efficiency, stability, mechanism and degradation pathway. Journal of Hazardous Materials 2019, 373, 85-96, doi:10.1016/j.jhazmat.2019.03.075.
4. Liu, J.; He, H.; Shen, Z.; Wang, H.H.; Li, W. Photoassisted highly efficient activation of persulfate over a single-atom Cu catalyst for tetracycline degradation: Process and mechanism. Journal of Hazardous Materials 2022, 429, 128398, doi:10.1016/j.jhazmat.2022.128398.

5. Cui, K.P.; He, Y.Y.; Xu, K.J.; Zhang, Y.; Chen, C.B.; Xu, Z.J.; Chen, X. Degradation of tetracycline hydrochloride by Cu-doped MIL-101(Fe) loaded diatomite heterogeneous Fenton catalyst. Nanomaterials 2022, 12, 811, doi:10.3390/nano12050811.
